# *In-vivo* screening implicates endoribonuclease *Regnase-1* in modulating senescence-associated lysosomal changes

**DOI:** 10.1007/s11357-023-00909-z

**Published:** 2023-08-29

**Authors:** Richard Venz, Anita Goyala, Abel Soto-Gamez, Tugce Yenice, Marco Demaria, Collin Y. Ewald

**Affiliations:** 1 Laboratory of Extracellular Matrix Regeneration, Institute of Translational Medicine, Department of Health Sciences and Technology, ETH Zürich, CH-8603 Schwerzenbach, Switzerland; 2 European Institute for the Biology of Aging (ERIBA)/University Medical Center Groningen (UMCG), Groningen, The Netherlands

**Keywords:** Senescence, Metformin, Longevity, Beta-gal, Senolytic

## Abstract

Accumulation of senescent cells accelerates aging and age-related diseases, whereas preventing this accumulation extends the lifespan in mice. A characteristic of senescent cells is increased staining with β-galactosidase (β-gal) ex vivo. Here, we describe a progressive accumulation of β-gal staining in the model organism *C. elegans* during aging. We show that distinct pharmacological and genetic interventions targeting the mitochondria and the mTORC1 to the nuclear core complex axis, the non-canonical apoptotic, and lysosomal-autophagy pathways slow the age-dependent accumulation of β-gal. We identify a novel gene, *rege-1/Regnase-1/ZC3H12A/MCPIP1*, modulating β-gal staining via the transcription factor *ets-4/SPDEF*. We demonstrate that knocking down *Regnase-1* in human cell culture prevents senescence-associated β-gal accumulation. Our data provide a screening pipeline to identify genes and drugs modulating senescence-associated lysosomal phenotypes.

**Supplementary Information:**

The online version contains supplementary material available at 10.1007/s11357-023-00909-z.

## Introduction

Cellular senescence is a biological phenomenon originally identified as the irreversible loss of replicative capacity (*i.e.,* Hayflick limit) [1, 2], accompanied by metabolic alterations, hypersecretion, and apoptosis inhibition [3, 4]. Cellular senescence covers pleiotropic functions by acting as a potent tumor suppressor mechanism and by playing a necessary role during development [5] and repair processes [6–8]. In contrast to these beneficial functions, senescent cells can become detrimental in aging tissues [9]. Beneficial and detrimental functions of senescent cells can be, at least in part, attributed to their persistence. Indeed, beneficial senescent cells are usually transient, whereas age-associated senescent cells persist and accumulate over time, promoting disease progression, organ dysfunction, and reduction of healthy lifespan [9–11]. While senescent cells naturally accumulate with aging, senescence states can also be induced by various extrinsic and intrinsic insults, irrespective of age [12].

Senescence-associated phenotypes are variable and include enlarged cell structure, expanded nucleoli, shortened telomeres, increased senescence-associated β-gal (SA-β-gal) activity, elevated levels of the cyclin-dependent kinase inhibitors (p21 or p16), metabolic dysfunctions, and a senescence-associated secretory phenotype (SASP) [13]. These salient features of senescence are exploited as biomarkers, but none of these markers taken individually are either specific or universal. Indeed, another layer of complexity is added by the fact that senescent cell populations are heterogeneous [14]. In mammalian cells, evaluation of the SA-β-gal staining has remained one of the gold standard biomarkers for identifying senescent cells, despite SA-β-gal activity results being dispensable for the maintenance of senescence states [11, 15, 16]. Although monitoring senescent-associated transgenic p21 markers in mice is possible [17], monitoring β-gal levels *in-vivo* and non-invasively during aging is not possible as tissues need to be harvested and processed before staining [18, 19]. This limitation can create biases in using SA-β-gal levels as a readout for interventions that interfere with senescent cell accumulation. On the other hand, drug screens in cell cultures have shown limited success since in vitro aging is different from *in-vivo* aging [20–22].

Here to bridge cell culture screening and *in-vivo* validation, we exploited the pioneering aging model organism *Caenorhabditis elegans.* With 60% homology to humans [23], several pathways that regulate the lifespan of this nematode (dies within 3–4 weeks) also regulate the mammalian lifespan [24, 25]. Because of its short lifespan, this nematode has been extensively used for genetic and drug screens [26]. A previously thought limitation was the fact that all somatic cells of *C. elegans* are post-mitotic and that only dividing cells undergo senescence. However, recent studies have demonstrated that post-mitotic cells can acquire senescence-associated phenotypes in mammals [27–29]. We confirm the previous findings [30] and show that β-gal staining increases with age in *C. elegans*. We show that by modulating mitochondrial longevity pathways, the progressive age-dependent increase of β-gal activity was postponed. Using a targeted genetic screening assay, we identify an RNA-binding protein with ribonuclease activities (*rege-1/*Regnase-1) that prevent age-related β-gal staining in both *C. elegans* and human cell cultures. Our approach to screen and identifying regulators of the beta-galactosidase activity enables the exploration of novel genetic and pharmacological interventions.

## Results

### Age-related increase of β-galactosidase staining in *C. elegans*

**Fig. 1:**
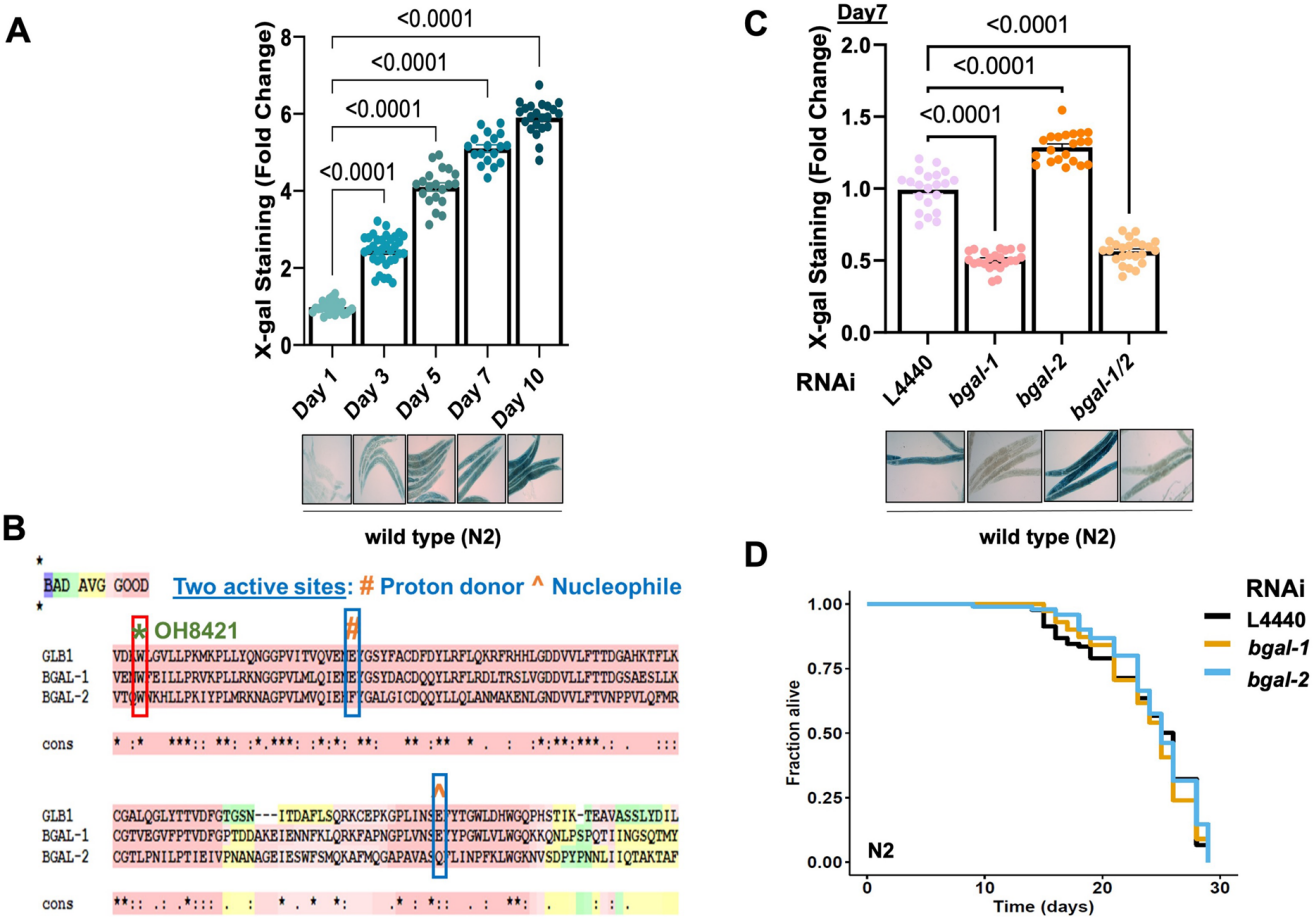
*bgal-1* is required for β-gal staining. **A** Bar graphs showed a linear increase in the β-gal staining in wild-type (N2) animals from day 1 adulthood to day 10. Representative images of stained N2 animals are shown below the graph. **B** Protein sequence alignment of BGAL-1 and BGAL-2 with the human homolog GLB1 showed conserved active sites (marked as ^ and #) in BGAL-1 and GLB-1. Nonsense mutation in OH8421, *bgal-1(ot594)* null mutant was shown as *. **C** Bar graph showed reduced β-gal staining upon *bgal-1* knockdown (KD) and a slight increase upon *bgal-2* KD. Double RNAi of *bgal-1* and *bgal-2* decreased the β-gal staining. Representative images of stained animals are shown below the graph. **D** Survival curve showed no change in the lifespan upon *bgal-1* or *bgal-2* RNAi

One of the most widely used biomarkers to identify senescent cells in tissues is the increased activity of the lysosomal senescence-associated beta-galactosidase (SA-β-gal) enzyme [11]. β-gal activities are assessed by providing the colorless substrate X-gal, which turns blue upon the activity of the enzyme at pH 4 [16] (Fig. S1A). However, because of the overactivity and expression of β-gal, senescent cells are able to convert the substrate at suboptimal pH 6 [31]. Interestingly, when we stained wild-type *C. elegans* with X-gal, we found a nearly linear increase in staining intensity with age, reaching its maximum intensity around day 10 of adulthood (Fig. 1A). In general, we noted that between days 10 and 12 of adulthood, the X-gal staining reached saturation. Time points after day 12 were not considered since *C. elegans* started to die then, and several stochastic deteriorative processes became visible [32–35]. To identify differences in age-related X-gal staining, throughout this study, we chose days 5 and 7 of adulthood, which reflect medium staining saturation allowing us to quantify lower and higher levels for comparison.

To ensure that our protocol reflects age-related β-gal, we examined three technical procedures that could lead to artifacts. First, the procedure for fixation includes freeze–thaw cycles to permeabilize *C. elegans* for X-gal to enter. We showed that the number of freeze–thaw cycles did not affect the staining intensity in animals (Fig. S1B). Second, staining did not happen if there was no X-gal added in the staining solution implying that it is the action of the β-gal enzyme on the X-gal in the animals that resulted in the blue end-product (Fig. S1C). Third, since we used FuDR to prevent progeny production and FuDR also affects protein homeostasis before and beyond the reproductive time period [36], we checked if FuDR had any effect on the staining efficiency. We found similar staining intensity between applying FuDR on wild type (N2) or using a temperature-sterile wild-type background mutant (*spe-9(hc88)*) (Fig. S1D).

### bgal-1 is required for the age-related β-gal staining but does not affect lifespan in *C. elegans*

The mammalian β*-*gal enzyme GLB1 has two orthologues in *C. elegans*, BGAL-1 and BGAL-2. Comparison of the protein sequences of GLB1 with BGAL-1 and BGAL-2 revealed around 50% similarity with each other, with maximum homology being in the glycoside hydrolase 35 catalytic domain (Fig. S1E). GLB1 has two active sites, a proton donor at residue 188 and a nucleophile at residue 268, which are conserved in *C. elegans’* BGAL-1, but not in *C. elegans’* BGAL-2 (Fig. 1B, S1E). To know whether the structural difference in BGAL-1 and BGAL-2 is translated into functional differences, we stained wild-type animals after knocking *bgal-1*, *bgal-2* or both. We observed β-gal enzymes in *C. elegans* operate opposite to each other, with *bgal-1* decreasing the staining while *bgal-2* increasing it (Fig. 1C). However, knocking both enzymes led to a decrease in staining, suggesting a genetic interaction between *bgal-1* and *bgal-2* with *bgal-1* being responsible for the β-gal staining in *C. elegans* (Fig. 1C). To validate these results, we used the non-sense mutant of OH8421 *bgal-1(ot594)*, which introduces a premature stop codon before the active site (Fig. S1E). As expected, there was no β-gal in mutant nematodes, further enforcing the idea that *bgal-1* is the functional β-gal enzyme required for the *β-*gal staining (Fig. S1F).

Although we observed a mild animal-to-animal variation in β-gal staining intensity, the staining within individual *C. elegans* was almost homogeneous. We wondered whether the blue end product could spread from animal to animal when *C. elegans* were permeabilized and fixed during the staining procedure. To test this, we stained day-7 adults of wild type (N2) and OH8421 *bgal-1(ot594)* mutants simultaneously together in the same preparation, reasoning that any blue stain observed in *bgal-1(ot594)* mutants should come from the wild-type animals that have the functional β-gal enzyme/BGAL-1 enzyme. Even in this setup, we did not observe any β-gal staining of OH8421 *bgal-1(ot594)* mutants (Fig. S1G). Similarly, co-staining young (day 1) and old (day 7) wild-type animals showed a faint staining for young and a strong staining for old *C. elegans* as characteristic of their age (Fig. S1 H). This implies that the blue end product was neither secreted nor shared among individuals and that this procedure faithfully measures the endogenous activity of the β-gal.

Given that senescent cells have higher levels of β-gal [11, 37], we asked if reducing β-gal levels could extend the lifespan and drive the aging process in reverse. Knocking either *bgal-1* or *bgal-2* did not affect the lifespan (Fig. 1D). Thus, similar to previous observations in mammalian cells, β-gal activity is dispensable for senescence-associated phenotypes, including potential pro-aging functions. Thus, β-gal reflects a biomarker of aging rather than a causal factor for aging.

### Accumulated β-gal staining reduced by senolytic and longevity-promoting drugs

**Fig. 2:**
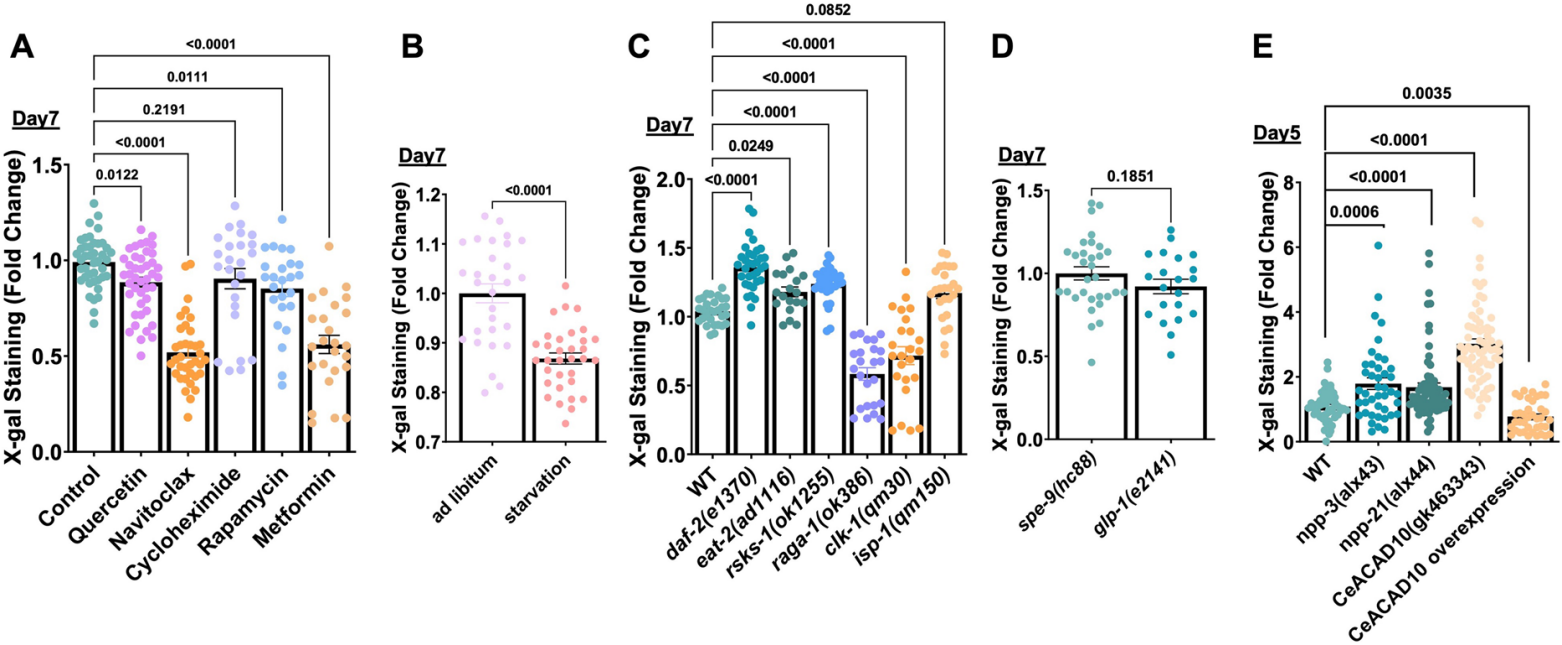
Interventions-specific suppression of β-gal staining. Bar graph showed (**A**) reduced β-gal staining in N2 animals treated with senolytics, quercetin & navitoclax, and longevity-inducing drugs, metformin & rapamycin, (**B**) reduced β-gal staining in starved animals, (**C**) reduced β-gal staining in *raga-1(ok386)* and *clk-1(qm30)* mutants, and slight increase in *daf-2(e1370)*, *eat-2(ad1116)* and *rsks-1(ok1255)* mutants, (**D**) no change in β-gal staining in the *glp-1(e2141)* mutant, (**E**) lower β-gal staining upon CeACAD10 overexpression at Day 5. A-D) The experiment was performed with Day 7 adult animals

Β-gal staining has been shown to be reduced using senolytic drugs [38, 39]. We, therefore, asked if the treatment of senolytic drugs like quercetin and navitoclax in *C. elegans* could reduce the β-gal staining. Quercetin is a flavonoid with a pleiotropic range of mechanisms of action, but its senolytic effects might be through the regulation of reactive oxygen species [38], and quercetin has been shown to increase the lifespan of *C. elegans* [40, 41]. Navitoclax (formerly ABT-263) is a Bcl-2 inhibitor that induces apoptosis in senescent but not in non-senescent cells [42]. We found that both drugs reduced the levels of β-gal staining at day 7 of adulthood compared to the control, with navitoclax showing the strongest effect (Fig. 2A). Since quercetin and navitoclax target the anti-apoptotic Bcl-2 family of proteins and, thus, might work through triggering the apoptotic pathway [43, 44], we next checked if the absence of the apoptotic checkpoint protein p53, *cep-1* in *C. elegans* could increase the β-gal staining. We did not observe any age-related β-gal staining change between wild type and p53/*cep-1(gk138)* mutants (Fig. S2A). In mammalian cells, senescence can be induced by UV [45, 46]. We UV-treated wild-type and *cep-1(gk138)* mutants but did not detect any change in β-gal staining (Fig. S2A). The age-dependent increase in β-gal staining in *C. elegans* might be, therefore, independent of the major mammalian UV damage-induced senescence pathway.

Next, we investigated whether longevity drugs, such as cycloheximide [47, 48], rapamycin [49], and metformin [50], would reduce the β-gal staining. While the biguanide drug metformin, commonly used to treat type 2 diabetes, decreased the staining drastically, comparable to navitoclax, the mTOR inhibitor rapamycin only slightly affected the β-gal staining. Cycloheximide, a protein translation inhibitor that only slightly extends lifespan [47, 48], also showed a decreasing trend, which is quite evident on day 5 of adulthood (Fig. 2A, S2B). Since mTOR and protein translation are reduced by dietary interventions, we starved animals for three days starting on day 4 of adulthood and found no difference at day 5 of adulthood (Fig. S2C) but a substantial reduction at day 7 of adulthood of the β-gal staining compared to ad libitum fed (Fig. 2B). These results suggest that reducing global protein translation might simply reduce the protein levels of the β-galactosidase enzyme, and this would result in lower staining intensity. Alternatively, slowing aging might postpone the accumulation of β-galactosidase activity.

### Selective longevity-promoting pathways prevent β-gal staining during aging

To gain mechanistic insights, we took a genetic approach by examining longevity-promoting mutants, such as Insulin/IGF-1 receptor mutant, *daf-2(e1370)*, dietary restriction-like mutant, *eat-2(ad1116)*, mTOR complex mutants, *raga-1(ok386)* and *rsks-1(ok1255)*, mitochondrial mutants, *clk-1(qm30)* and *isp-1(qm150),* and germline mutant, *glp-1(e2141)* (for which we used respective control, *spe-9(hc88))*, to determine the effect of these interventions on β-gal staining. We found that *raga-1(ok386)* and *clk-1(qm30)* mutants showed a significant reduction in the β-gal staining, both at adult day 7 and day 5, while all other mutants showed a slight increase in the staining at adult day 7 (Fig. 2C, S2D). *glp-1(e2141)* mutation did not change the staining intensity (Fig. 2D). To ensure we assess the staining intensity at the correct time points, we performed a time course analysis of β-gal staining with *daf-2(e1370)*, *eat-2(ad1116),* and *glp-1(e2141)* mutants, which showed an age-dependent and progressive increase in β-gal staining intensity (Fig. S2 E, F). This suggests that simply slowing aging does not result in a lesser accumulation of β-gal staining. Furthermore, both *daf-2(e1370)* and *eat-2(ad1116)* reduce global protein translation [51, 52], suggesting that the role of protein translation in reducing β-gal staining is minimal. Although both *raga-1* and *rsks-1* are associated with mTOR complex 1 and regulate global protein translation [48, 49, 52, 53], only *raga-1* but not *rsks-1* inhibition was sufficient to reduce β-gal staining (Fig. 2C, S2D), suggesting a more specific molecular requirement. Taken together, not all longevity interventions slow the accumulation of β-gal staining. In particular, we identified interventions that affect mitochondrial longevity pathways, such as metformin and *clk-1* but not *isp-1* (Fig. 2C, S2D), and mTOR complex 1 inhibition, such as rapamycin and *raga-1* but not *rsks-1* (Fig. 2C, S2D), to be able to postpone β-gal staining.

Recently, the action of metformin on mitochondria was shown to be mediated by the nuclear pore complex (NPC)-mTORC1-acyl-CoA dehydrogenase family member 10 (ACAD10) genetic axis [54]. Also, inactive RAGA-1 was reported to be the dominant negative inhibitor of mTORC1 [55, 56]. Since β-gal staining was reduced in *raga-1(ok386)* mutant, rapamycin- and metformin- treatment, all of which were implicated in this NPC-ACAD10 longevity-promoting axis, we investigated if the response elements- NPC and CeACAD10 are also required for regulation of β-gal. We tested β-gal staining in the central core component, *npp-3(alx43)* and nuclear basket component, *npp-21(alx44)* mutants of NPC, and CeACAD10*(gk463343)* mutants at adult day 5. We found a slight increase in β-gal staining of NPC mutants compared to wild type (Fig. 2E, S2G). While CeACAD10(*gk463343)* mutants showed increased staining, overexpression of CeACAD10 was sufficient to reduce β-gal staining (Fig. 2E, S2G). Although there might be a role of the NPC-ACAD10 longevity-promoting axis, we currently only have tantalizing evidence pointing us in this direction (Fig. S2H).

### A targeted screen to identify the modulator of β-gal accumulation

Given that not all longevity-promoting pathways were able to postpone the β-gal staining in *C. elegans*, we sought to identify the modulators of β-gal accumulation in *C. elegans* by employing a targeted screen of a variety of mutants and RNAi knockdown. Since the Bcl-2 inhibitor navitoclax drives senescent cells into apoptosis, we targeted the *C. elegans* apoptosis pathway (Fig. S3A). Reduction of function mutation in the Bcl-2 homolog *ced-9(n1950) *[57] phenocopied the lower β-gal staining of navitoclax (Fig. S3 B). However, loss of *egl-1/*BH3, the negative regulator of *ced-9*/Bcl-2, also showed reduced β-gal staining during older age (Fig. S3 B), the opposite result expected from the genetic pathway (Fig. S3 A). Furthermore, *ced-9*/Bcl-2 is the negative regulator of *ced-4*, which activates the caspase *ced-3* required for apoptosis [58–64]. However, we detected no difference in the β-gal staining of *ced-4(n1162)* mutants compared to wild type (Fig. S3 B), suggesting that the reduction in β*-*gal staining caused by a mutation in *egl-1/*BH3 or *ced-9*/Bcl-2 are mediated independently of the canonical apoptosis pathway.

Because the β-gal enzyme resides in the lysosomes, we screened mutants related to enzymes in lysosomes (*spp-10*), proteins involved in lysosome biogenesis (*glo-1*, *glo-3*, *glo-4*), vacuolar pumps maintaining the acidity inside lysosomes (*vha-2*, *vha-3*, *vha-12*), proteins important for lysosomal integrity (*scav-3*), lysosome-related organelles-gut granules (*glo-1,3,4*) and a lysosome-related process-autophagy mutant (*unc-51*). Amongst all these, only the sphingolipid-altering saposin *spp-10(gk410)* mutant showed reduced β-gal staining, whereas mutations in the guanine nucleotide exchange factor *glo-4*, required for the Rab-like GTPase *glo-1* [65, 66] and autophagy-related kinase *unc-51*/ATG1, increased the age-related β-gal staining (Fig. S3 B). Interestingly, GLO-4 physically binds RPM-1 [66], and RPM-1 inhibits UNC-51/ATG1 activity and autophagosome formation [67], suggesting a role of lysosome-related autophagy in preventing the accumulation of β-gal staining.

**Fig. 3:**
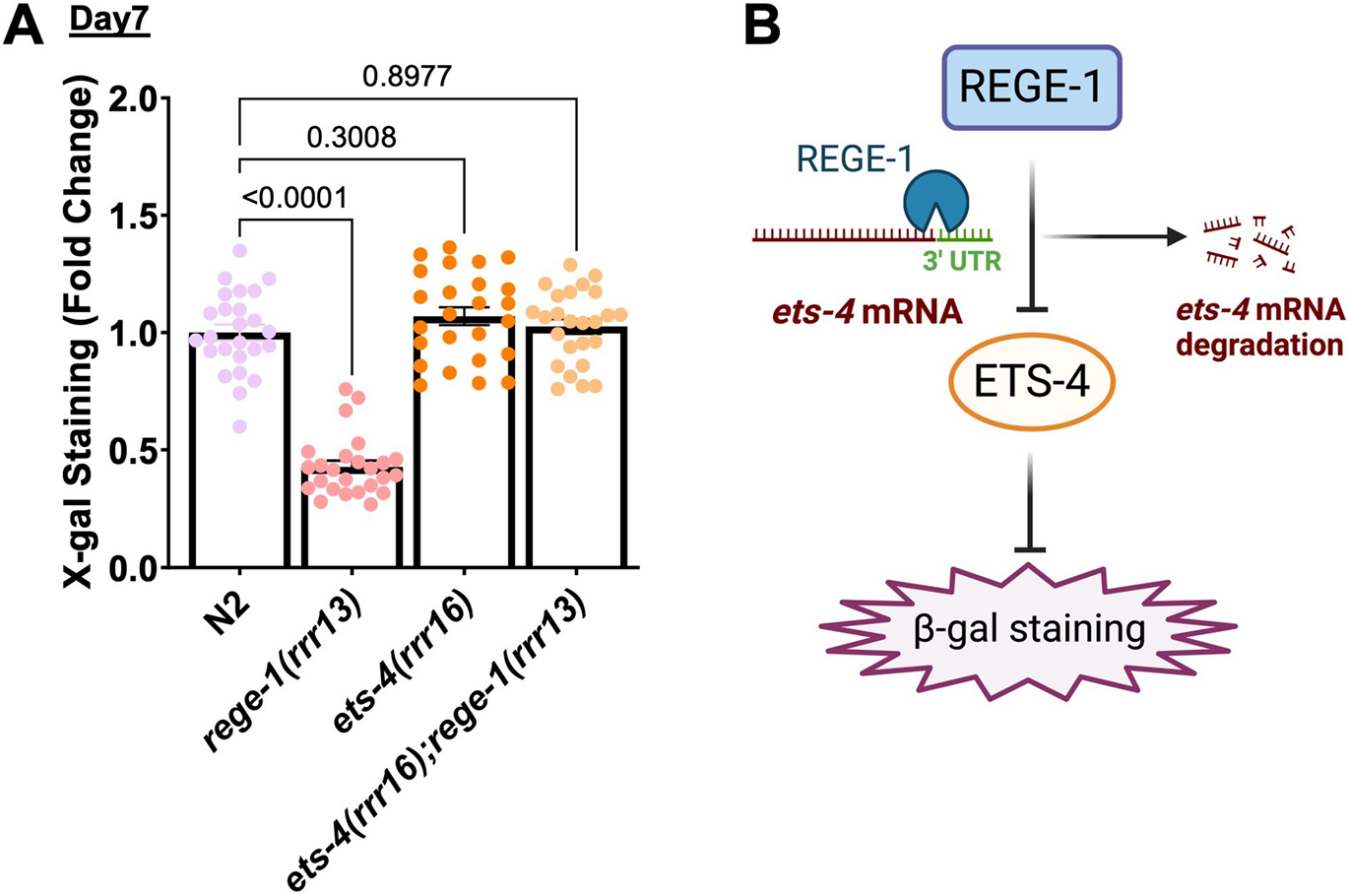
Regulation of β-gal staining by *rege-1*. **A** Bar graph showed reduced β-gal staining in the *rege-1(rrr13)* mutant, which was reversed by *ets-4* mutation. **B** Model showing the regulation of *ets-4* mRNA degradation and, thus, β-gal activity changes by REGE-1

Because mutations in *spp-10* alter sphingolipid and potentially lipid bilayers on lysosomes and endoplasmic reticulum, we knocked down genes in the sphingolipid and ceramide biosynthesis pathway as well as general regulators of lipid metabolism and ER stress (Fig. S3C). The strongest suppressor of progressive accumulation β-gal staining during aging was the endoribonuclease *rege-1* (Fig. S3C). We confirmed these results of reduced levels of β-gal staining with *rege-1(rrr13)* mutants lacking the RNAase domain [68] (Fig. 3A, S3D). The transcription factor ETS-4 was previously identified as the main target of REGE-1 [68] (Fig. 3B). Mechanistically, the RNase *rege-1* binds the 3’UTR and induces degradation of the *ets-4* mRNA, thereby controlling ETS-4 levels and preventing its transcriptional output (Fig. 3B) [68]. Therefore, we assessed the β-gal staining in *ets-4(rrr16);rege-1(rrr13)* double mutants and found that β-gal staining levels were restored to wild-type levels at day 7 of adulthood (Fig. 3A, S3D).

Taken together, our target screen identified a regulatory system of an RNAse and transcription factor, REGE-1 and ETS-4, that has not yet been implicated in β-galactosidase accumulation (Fig. 3B).

### Mammalian Regnase-1 regulates senescence-associated lysosomal changes in culture

**Fig. 4:**
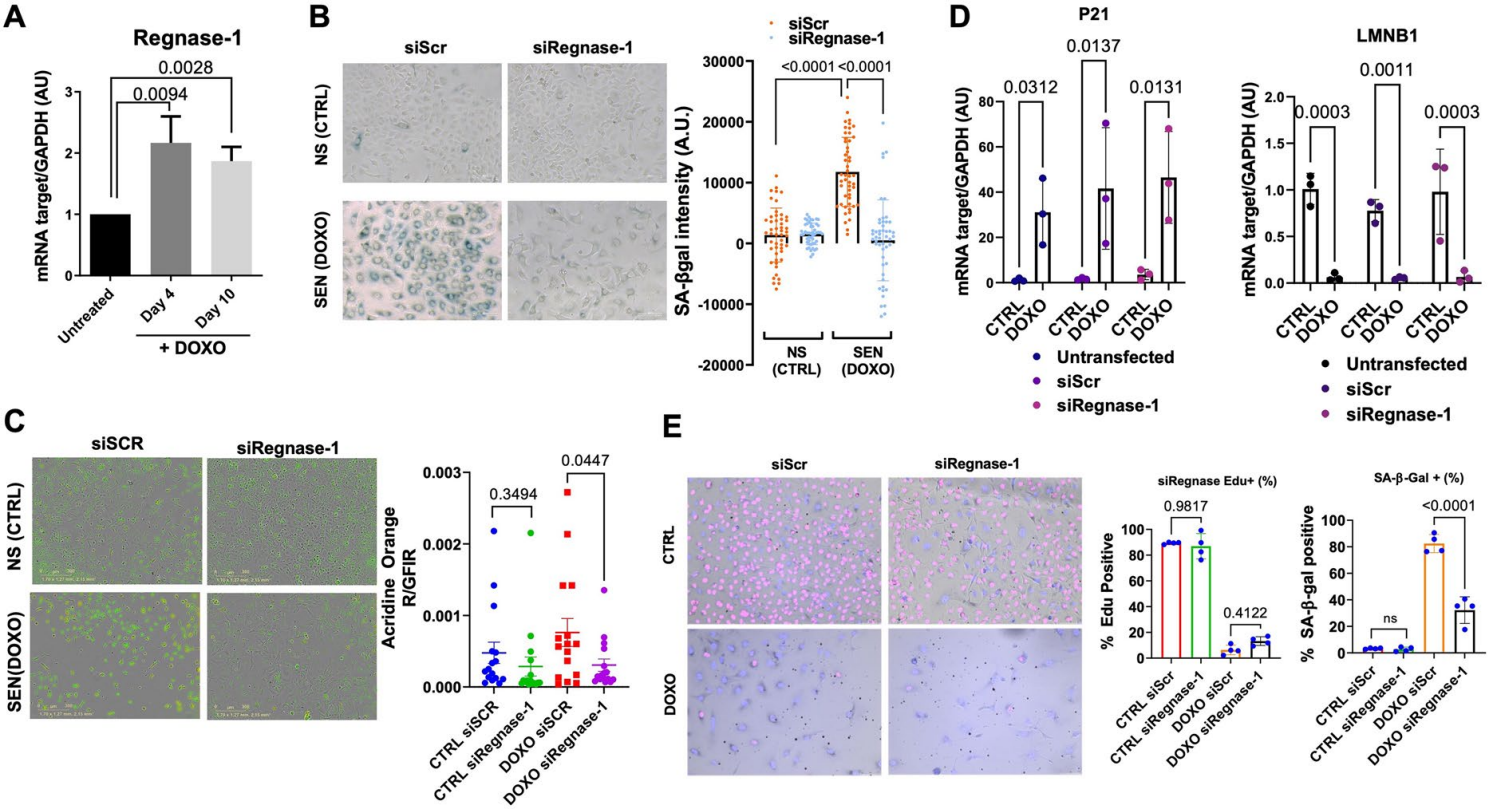
**A** Bar graph showing upregulated Regnase-1 mRNA levels in doxorubicin treated cells (DOXO) at day 4 and day 10 after treatment (mean fold change with SD relative to untreated control is shown, from *n* = 3). **B** Representative images and quantification showing reduced SA-β-gal staining intensity in DOXO-treated A549 cells upon silencing Regnase-1 (graph shows the average intensity of different pictures from at least three independent experiments)*.* **C** Representative images and quantification showing reduced Acridine Orange staining in DOXO-treated A549 cells upon silencing Regnase-1*.* The ratios of red-to-green fluorescence intensity are shown. For high-resolution images and details, please see Supplementary Fig. S4D. **D** mRNA levels of P21(CDKN1A) and lamin B1 (LMNB1) in siRNA/DOXO treated A549 cells relative to untreated (CTRL) / untransfected controls (mean + SD, from *n* = 3 is shown). **E** Representative images of co-stained SA-β-gal staining and incorporated EdU in siRNA-treated A549 cells. The percentage of SA-β-gal staining positive cells, or EdU positive cells, is shown (mean + SD, *n* = 4)

To determine if *rege-1* is a conserved regulator of β-gal in human cells, we first determined whether the mRNA expression of the human orthologue of *rege-1* called Regnase-1 (former name *MCPIP1*), encoded by the *ZC3H12A* gene [69], is altered in senescent cells. We used doxorubicin to induce senescence in adenocarcinomic human alveolar basal epithelial cells (A549) and validated senescence by qRT-PCR of genes involved in senescence, namely p21, MMP-1, IL-8, and LMNB1 (Fig. S4A). We found that after 4 and 10 days of doxorubicin treatment, Regnase-1 mRNA was increased two-fold (Figs. 4A, S4B, C), suggesting that Regnase-1 levels increase when cells become senescent. To determine whether this increase of Regnase-1 expression has a functional role in regulating SA-β-gal, we knocked down Regnase-1 by small interfering RNA (siRegnase-1) in A549 cells (Fig. S4B). Interestingly, siRegnase-1 in doxorubicin-induced senescent cells completely suppressed β-gal staining (Fig. 4B). To evaluate whether Regnase-1 could revert additional senescence-associated properties, we measured various senescence readouts. First, we used a ratiometric analysis using Acridine Orange staining, which increases linearly with acidic organelle/lysosomal volume but is not affected by differences in cytoplasmic RNA or changes in the cellular volume, which are characteristic of senescent cells. We observed an increased acridine orange staining in doxorubicin-treated senescent cells, but the levels were reduced after interfering with Regnase-1 (Figs. 4C, S4D, E). Second, we measured if Regnase-1 levels could influence p21 expression. However, we did not observe any reduction in p21 in siRegnase-1 senescent cells (Fig. 4D). Finally, we monitored cell cycle arrest and did not observe any rescue of EdU incorporation in senescent cells carrying siRegnase-1 (Fig. 4E). Thus, our results demonstrate the functional role of Regnase-1 in promoting senescence-associated β-gal and generally lysosomal activity in human senescent cells with no impact on senescence-associated growth arrest mechanisms.

## Discussion

*In-vitro* screening, molecular, and systems biology approaches have implicated about 300 genes in senescence [70]. To facilitate the discovery of genes and molecular underpinning, we reestablished β-gal staining for *C. elegans* to discover and bridge findings to mammals. We found that the β-gal staining almost linearly increased early during aging, plateauing post-reproductively by days 10–12 of adulthood, a time point where *C. elegans* tissues start to stochastically deteriorate, and first deaths occur. Prohibiting this β-gal accumulation via knocking down the β-gal enzyme had no effect on lifespan. This suggests that accumulation in β-gal staining correlates with age rather than a causally implicated process driving *C. elegans* aging. Our study does not provide evidence to conclude whether the β-gal staining is specific for senescent cells nor whether *C. elegans* has senescent cells as observed in mammals. However, our data point toward the idea that the β-gal staining reflects lysosomal content changes in *C. elegans*, in line with our findings using human cell culture and reported findings by other groups investigating mammalian senescent cells using β-gal staining and Acridine Orange [71].

Consistent with this idea and the underlying process of the observed accumulation in β-gal staining is not a causal driving factor *C. elegans* aging is the observation that not all longevity interventions slowed this progressive increase of β-gal staining. Using pharmacological and genetic approaches, our data suggest distinct pathways implicating mitochondria and lysosomal signaling (Fig. S2H). Furthermore, even interventions that target the mitochondria to extend lifespan, such as reducing the function of mitochondrial hydroxylase *clk-1/Mclk1* important for ubiquinone synthesis or *isp-1*, the mitochondrial complex III subunit gene, showed a reduction in β-gal staining or not, respectively. Similarly, extending lifespan by reducing mTOR complex 1 (mTORC1) function by either rapamycin or *raga-1(ok386)* mutants slowed the age-dependent accumulation of β-gal staining. By contrast, blocking downstream *rsks-1*/S6 kinase of mTORC1 signaling did not alter β-gal staining.

Adding to these results are the findings that the Bcl2 inhibitor Navitoclax and EGL-1, a molecular inhibitor of Bcl2, or directly inhibiting CED-9/Bcl2 lowered the age-dependent β-gal staining but not any other downstream apoptotic genes or upstream interventions, such as targeting p53/CEP-1. The observed effects may be, therefore, independent of the role of CED-9/Bcl2 in inhibiting the apoptotic pathway and relate instead to its other molecular functions. For instance, CED-9/Bcl-2 can directly interact with the evolutionarily conserved autophagy protein Beclin1, thereby inhibiting autophagosome formation and autophagy [72]. Interestingly, CED-9/Bcl2 also localizes to the outer mitochondrial membrane [73] and has recently been implicated as being important for mitochondrial fission and fusion [74], thereby linking mitochondrial dynamics to β-gal staining (Fig. S2H). Recently in mammals, mitochondrial dysfunction can drive cells into senescence (MiDAS) [75], and senolytic inhibition of Bcl2 changes mitochondrial membrane potential and thereby promoting necrosis in addition to apoptosis [76], suggesting a conserved non-canonical link to our findings in *C. elegans*. Interestingly, in our selected pharmacological screening approach, we also identified metformin, a biguanides anti-diabetic drug, and its predecessor, phenformin, has been shown to alter mitochondrial dynamics for promoting longevity in *C. elegans *[77]. Given these sub-pathways-specific findings (Fig. S2H), how would these observations connect together?

Strikingly, a study investigating growth inhibition of metformin in cancer cells and *C. elegans* identified a unified mechanism linking metformin, mitochondria, *raga-1*/RagA, to mTORC1 [54]. We hypothesized that this mechanism might connect all the seemingly loose dots of our β-gal staining results. To test this, we investigated key components of this mechanism, including inhibiting nuclear core complex components and the downstream metformin effector ACAD10 (Acyl-coA dehydrogenase family member 10) (Fig. S2H). Metformin increases ACAD10 levels via nuclear shuttling of RagA/C to mTORC1 [54]. Consistent with this model and our findings with metformin lowering the age-dependent β-gal staining, loss of CeACAD10 showed increased while overexpression of CeACAD10 showed decreased β-gal staining. Therefore, we conclude that in *C. elegans,* specific longevity interventions centering around the mitochondria-mTORC1 axis ameliorate the age-dependent accumulation of β-gal staining.

Furthermore, RagA/C mediates the translocation of mTORC1 to the lysosome [78], which is important for downstream signalings, such as regulating autophagy. In our screen, we also identified components involved in lysosomal-autophagy pathways that, when lost, aggravate the age-dependent increase of β-gal staining. Importantly, interventions that enhance or promote the pathway leading to lysosomal-autophagy, such as starvation, rapamycin [49], metformin [79], or navitoclax [80], resulted in a decrease of age-dependent β-gal staining, similar to what was observed in disrupted autophagy in *raga-1(ok386)* mutants. On the contrary, mutations that result in reduced autophagy, such as *unc-51(e369)* [67], or perhaps *glo-4(ok623),* resulted in increased β-gal staining. Because increased lysosomal content is considered a hallmark of senescence of which, SA-β-gal staining is used as a surrogate marker [77]. Our findings suggest that selective autophagy may help prevent the accumulation of age-dependent β-gal staining, possibly due to lysosomal degradation via autophagy, a process also known as lysophagy.

Perhaps the most interesting aspect is that we were able to translate genetic findings to human cellular senescence. We identified the mRNA-binding endoribonuclease *rege-1*/Regnase-1 as a mediator of age-dependent accumulation of β-gal staining in *C. elegans*. REGE-1 regulates innate immunity stress response and fat metabolism in *C. elegans* [68, 81]. Similarly, in mammals, Regnase-1 regulates the rapid response to stress and inflammation [82]. Interestingly, Regnase-1 is activated by processes implicated in senescence, such as IL-1β, TNF-alpha, LPS, S100A8/9, HMGB1, and NFκB, on its promoter region and also by posttranslational regulations, such as IKKs/IRAK1 [83], suggesting parallels or implicating Regnase-1 responding to senescence. Regnase-1 targets specific mRNAs for degradation by recognizing a specific region in the 3’ UTR of the target mRNA [84]. However, Regnase-1 can also act as a transcriptional co-activator with its zinc finger domain [85] or as a deubiquitinase, for example, on NEMO [86] and TRAF6 [87, 88]. Not surprisingly, Regnase-1 has multiple physiological functions, including T-cell and monocyte activation, apoptosis, cell differentiation, angiogenesis, and adipogenesis [82, 89]. And therefore is also implicated in many pathologies, such as promoting rheumatoid arthritis, psoriasis, atherogenesis, and ischemia, but preventing or inhibiting autoimmune gastritis, anemia, and carcinogenesis [82, 90–93]. Regnase-1 knockout mice die within three months after birth [94–96], and tissue-specific knockout results in a short lifespan [97–99]. By contrast, inhibiting Regnase-1 in T cells, reprograms these T cells into long-lived cytotoxic CD8 effector cells that directly kill tumor cells [100], thereby suggesting a protective role of Regnase-1 during aging. Thus, Regnase-1 inhibition might be a double-edged sword for aging, depending on the tissue. Although we show that inhibition of Regnase-1 interferes with SA-β-gal and lysosomal activities in human senescent cells, further studies are needed to determine the role of Regnase-1 in mammalian senescence.

Moreover, how mammalian Regnase-1 reduces lysosomal activity, including SA- β-gal, remains to be determined. Potentially, Regnase-1 could target longevity-associated transcription factors C/EBPβ [101], BATF [100, 102], and SPDEF [81]. It is known that Regnase-1 binds 3’UTR of C/EBPβ and BATF mRNA and promotes its degradation [100, 103]. The *C. elegans* transcription factor ETS-4, the orthologue of SPDEF, is a direct target of *rege-1* and is implicated in regulating lifespan [68, 81]. In human prostate cancer patients, the SAM pointed domain containing ETS transcription factor (SPDEF) was recently identified as a novel transcriptional regulator of the cellular senescence-related gene prognostic index (CSGPI) [104]. Furthermore, high SPDEF expression is favorable in endometrial cancer [105] (Fig. S4 F (The Human Protein Atlas, version 21.2 https://www.proteinatlas.org/ENSG00000124664-SPDEF/pathology/endometrial+cancer)). Similarly, forcing the expression of SPDEF suppresses tumorigenesis [106]. Interestingly, SPDEF binds to the promoter of FOXM1, which in a feedback loop is involved in cancer [107, 108]. Although chronic SPDEF and FOXM1 can promote or inhibit cancer, cyclic FOXM1 activation has recently been shown to increase the lifespan of mice [109]. Although these observations are tantalizing, the role of Regnase-1 and its potential targets in senescence need to be investigated in future work.

In summary, our data show that longevity interventions targeting the ancient and potentially conserved mitochondria-mTORC1 axis aggravate the accumulation of β-gal staining and establish *C. elegans* as a new *in-vivo* surrogate system for senescence-related β-gal changes. As a proof-of-concept, we identified a novel gene, *rege-1*/Regnase-1, to prevent age-dependent β-gal accumulation in *C. elegans*, and also block β-gal accumulation in human cells. We demonstrate that REGE-1 mechanistically works via ETS-4/SPDEF transcription factor to modulate β-gal staining in *C. elegans*. This regulatory mechanism opens up new investigations and implications of Regnase-1 in potentially preventing the accumulation of pathological senescent cells in mammals and ultimately provides a strategy to promote health during aging.

## Materials and Methods

### Strain maintenance

All strains were maintained at 20 °C except *spe-9(hc88)* and *glp-1(e2141)* mutants which were maintained at 15 °C. The animals were grown on NGM (nematode growth media) plates and fed with *Escherichia coli* OP50. The strains used in this study are: N2 (wild-type Bristol), TJ1060 [*spe-9(hc88)*], OH8421 [*bgal-1(ot594)*], BC13743 [*dpy-5(e907)* I; sEx13743], CB1370 [*daf-2(e1370)*], DA1116 [*eat-2(ad1116)*], RB1206 [*rsks-1(ok1255)*], VC222 [*raga-1(ok386)*], MQ130 [*clk-1(qm30)*], MQ887 [*isp-1(qm150)*], CB4037 [*glp-1(e2141)*], *npp-3(alx43)*, *npp-21(alx44)*, *CeACAD10(gk463342)*, CeACAD10 Overexpression, TJ1 [*cep-1(gk138)*], MT8735 [*egl-1(n1084n3082)*], MD792 [*ced-13(sv32)*], MT4770 [*ced-9(n1950)*], MT2547 [*ced-4(n1162)*], RB1227 [*scav-3(ok1286)*], RB807 [*vha-2(ok619)*], VC1003 [*vha-3(ok1501)*], RB938 [*vha-12(ok821)*], JJ1271 [*glo-1(zu391)*], RB811 [*glo-4(ok623)*], GH403 [*glo-3(kx94)*], VC749 [*spp-10(gk410)*], CB369 [*unc-51(e369)*], *rege-1(rrr13)*, *rege-1(rrr13); ets-4(rrr16)*, and *ets-4(rrr16)*.

### β-Galactosidase (β-gal) staining in *C. elegans*

#### Fixation

For fixation of animals, the plates were washed off in M9 buffer containing Tween-20 (0.1%) and collected into 1.5 ml microcentrifuge tubes. Animals were allowed to settle down and then washed three times with M9 buffer. After the last wash, the supernatant liquid was removed maximally. An equal volume of 8% paraformaldehyde (approx. 30 uL) was added to the residual liquid to achieve a final conc. of 4% fixation solution. Following an incubation period of 30 s at room temperature, samples were frozen in liquid nitrogen. Fixed samples can now be stored in the -80 °C freezer for later use.

#### X-Gal staining solution preparation

X-Gal staining solution was prepared following instructions of the staining kit (Senescence β-Galactosidase Staining Kit, #9860 Cell Signaling Technology). For each mL of X-Gal Staining Solution, 930 µl of 1X staining buffer (837 µl of ddH2O + 93 µl of 10X staining buffer), 10 µl of solution A, 10 µl of solution B, and 50 µl of X-Gal solution (20 mg/ml X-Gal dissolved in DMF) were mixed in a 15 mL falcon tube. The optimal pH of the solution was measured to be 5.5–6.0. It is important to note here that two variables, pH and DMF played a role in determining the staining. In particular, staining was optimal when pH of the staining solution was 5.5–6.0, and X-gal should be dissolved in DMF from Sigma with the highest purity ≥ 99% (#D4551, Sigma). Also, only the X-gal provided in the kit works best for this staining protocol (we tried X-gal from Roche #10,651,745,001).

#### Staining

The frozen samples were thawed at room temperature for 10 min before again flash freezing them in liquid nitrogen. The freeze–thaw was repeated for up to four cycles. Then, the samples were washed with 1 mL PBS pH 6.0 & 200 µl of PBS-Tween 20 (0.1%) buffer three times. The supernatant liquid was discarded completely, and 150 µl of X-Gal staining solution was added. The samples were incubated overnight on the rotor at 37 °C. Following overnight staining, samples were then washed once with 1 ml of PBS pH 6.0 and 200 µl of PBS-Tween (% 0.1) buffer and then mounted on 2% agarose pads for microscopy.

#### Imaging and analysis

The animals were visualized at 10X using a brightfield microscope (Tritech Research). NIH ImageJ software was used to quantify staining in the animals; Images were split into three color channels, and red color channel intensity was quantified for the analysis. Statistical analysis of the quantified data was performed using One-way ANOVA and t-test in GraphPad Prism.

## Lifespan Assay

Gravid adult wild-type animals were treated with hypochlorite solution to obtain L1 synchronized population. These were grown on OP50 seeded NGM plate till the L4 stage. At the L4 stage, 50 animals were transferred to RNAi NGM plates containing FuDR, seeded with corresponding bacteria (L4440/*bgal-1*/*bgal-2*) in replicates of four plates. Survival of the animals was scored by prodding their tail once with platinum wire every alternate day. Unhealthy animals showing vulval bursting or those crawling to the sides/lid of the plates were censored from the population. The lifespans were performed in two biological replicates at 20 °C. Statistical analysis was performed using JMP.

## *In-vitro* senescence assays

The human lung epithelial carcinoma cell line A549 (ATCC®, CCL­185™) was cultured in RPMI supplemented with GlutaMax (Gibco, Ref. 61,870–010, USA) and 10% FBS and 1% Penicillin/Streptomycin (Gibco, Ref. 15,140–122). Cellular senescence was induced by treating A549 cells with 250 nM doxorubicin hydrochloride (Tebu-bio, Ref. BIA-D1202-1, France) for 24 h previously described [110]. After treatment, culture media was refreshed every 48 h. At days 1, 4, and 10 after doxorubicin treatment, cells were fixed in 2% formaldehyde and 0.2% glutaraldehyde or collected in an RLT buffer and stored at -80 °C until qPCR analysis. Fixed cells were stained for SA-β-gal activity and EdU incorporation.

## Regnase-1 siRNA

Regnase-1 (ZC3H12A) siRNAs (Ref siRNA ID: s36969**;** Thermo Fisher Scientific) were used to knockdown Regnase-1 levels in A549 cells. Cells were seeded in 96 or 24 multi-well plates, 24 h after seeding, cells were transfected with Regnase-1 siRNAs or negative control siRNA (Silencer™ Select Negative Control No. 1 siRNA. Ref. 439,084; Thermo Fisher Scientific) using Lipofectamine™ RNAiMAX transfection reagent (Ref. 13,778,100; Thermo Fisher Scientific) following manufacturer instructions. For senescence induction, cells were treated with 250 nM doxorubicin in fresh culture medium 48 h after transfection. The culture medium containing doxorubicin was replaced 24 h after treatment for fresh media without doxorubicin. Regnase-1 downregulation was confirmed 2 days after transfection. For SA-β-gal activity, cells were fixed in 2% formaldehyde, 0.2% glutaraldehyde 4 days after doxorubicin treatment when SASP markers have increased, and cells are positive for SA-β-gal.

## Acridine orange ratiometric analysis for acidic organelles

Acridine orange staining was adapted as previously described by Thomé et al., 2016 [111]. Briefly, live cells were stained in culture media containing 2 µM acridine orange for 15 min at 37 °C; following incubation, cells were imaged using an Incucyte Zoom® live-cell analysis system collecting data from phase contrast and both green and red fluorescence (400 ms, and 800 ms, respectively). Fluorescence intensities from each channel were measured for all samples using the IncuCyte Zoom 2018A software, and the red-to-green fluorescence intensity ratio (R/GFIR) was calculated for each sample using pooled data from 16 images per condition, and a higher R/GFIR ratio indicating higher lysosomal volume.

### Supplementary Information

Below is the link to the electronic supplementary material.

Supplementary file1 (JPEG 1521 KB)

Supplementary file2 (JPEG 1232 KB)

Supplementary file3 (JPEG 920 KB)

Supplementary file4 (JPG 991 KB)

## Data Availability

All data is provided in the supplementary material.
