# Supplementary figures and images for: In-vivo screening implicates endoribonuclease Regnase-1 in modulating senescence-associated lysosomal changes
Source: GeroScience. 2023 Aug 29;46(2):1499–514. doi: 10.1007/s11357-023-00909-z (PMC10828269; doi:10.1007/s11357-023-00909-z)

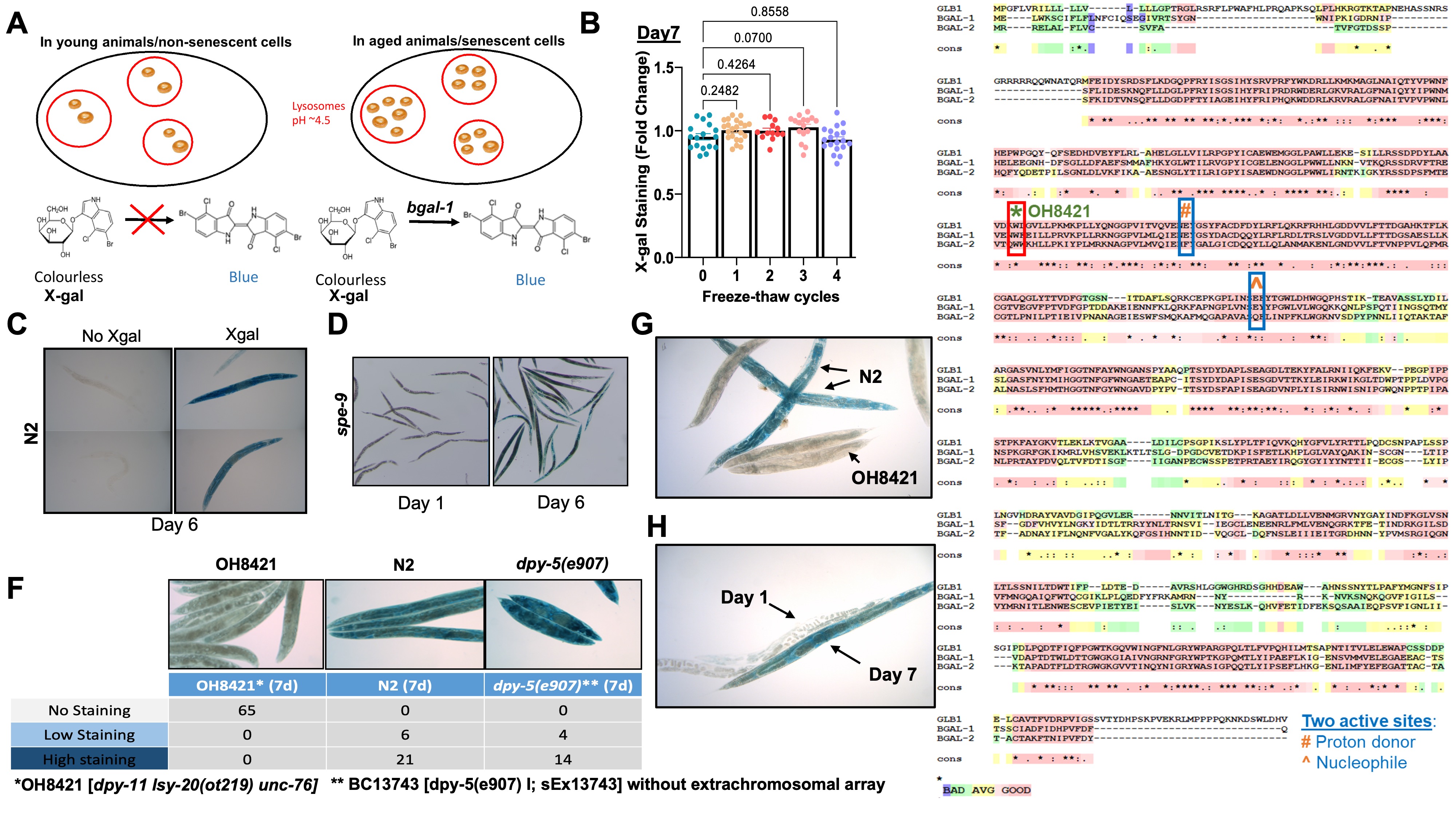

Supplement: Supplementary file 1 — Supplementary file1 (JPEG 1521 KB) [file 11357_2023_909_MOESM1_ESM.jpeg]

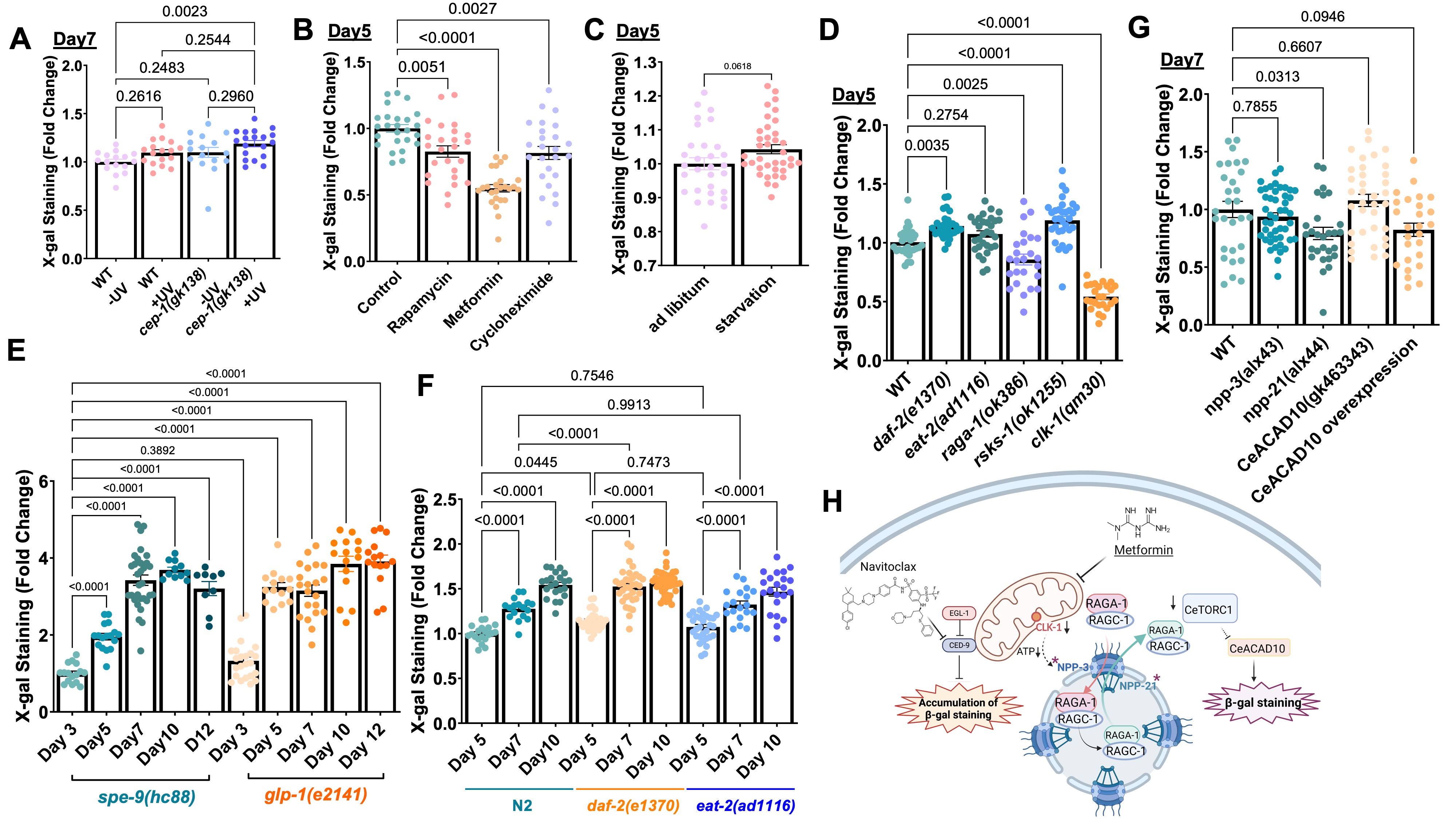

Supplement: Supplementary file 2 — Supplementary file2 (JPEG 1232 KB) [file 11357_2023_909_MOESM2_ESM.jpeg]

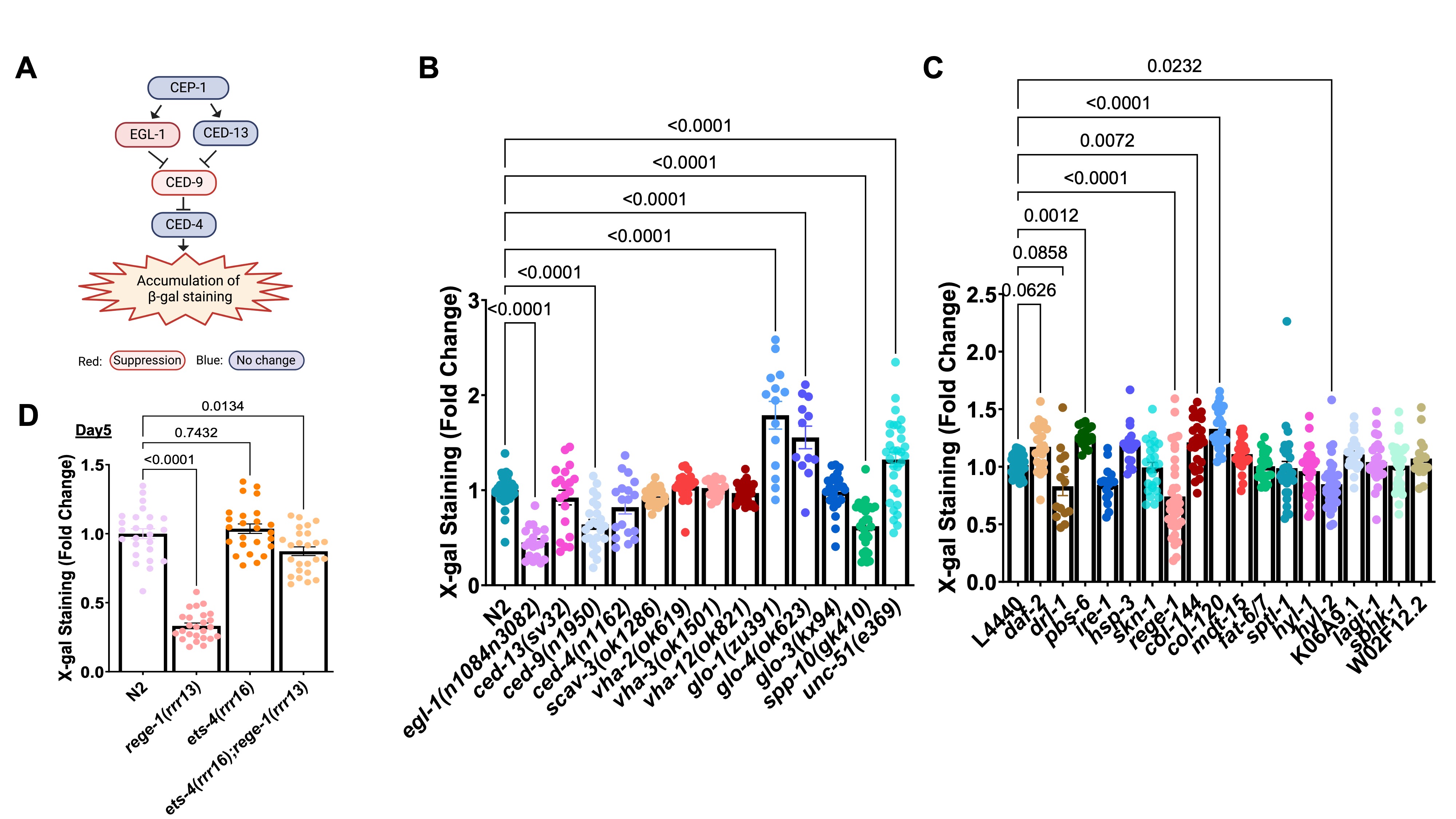

Supplement: Supplementary file 3 — Supplementary file3 (JPEG 920 KB) [file 11357_2023_909_MOESM3_ESM.jpeg]

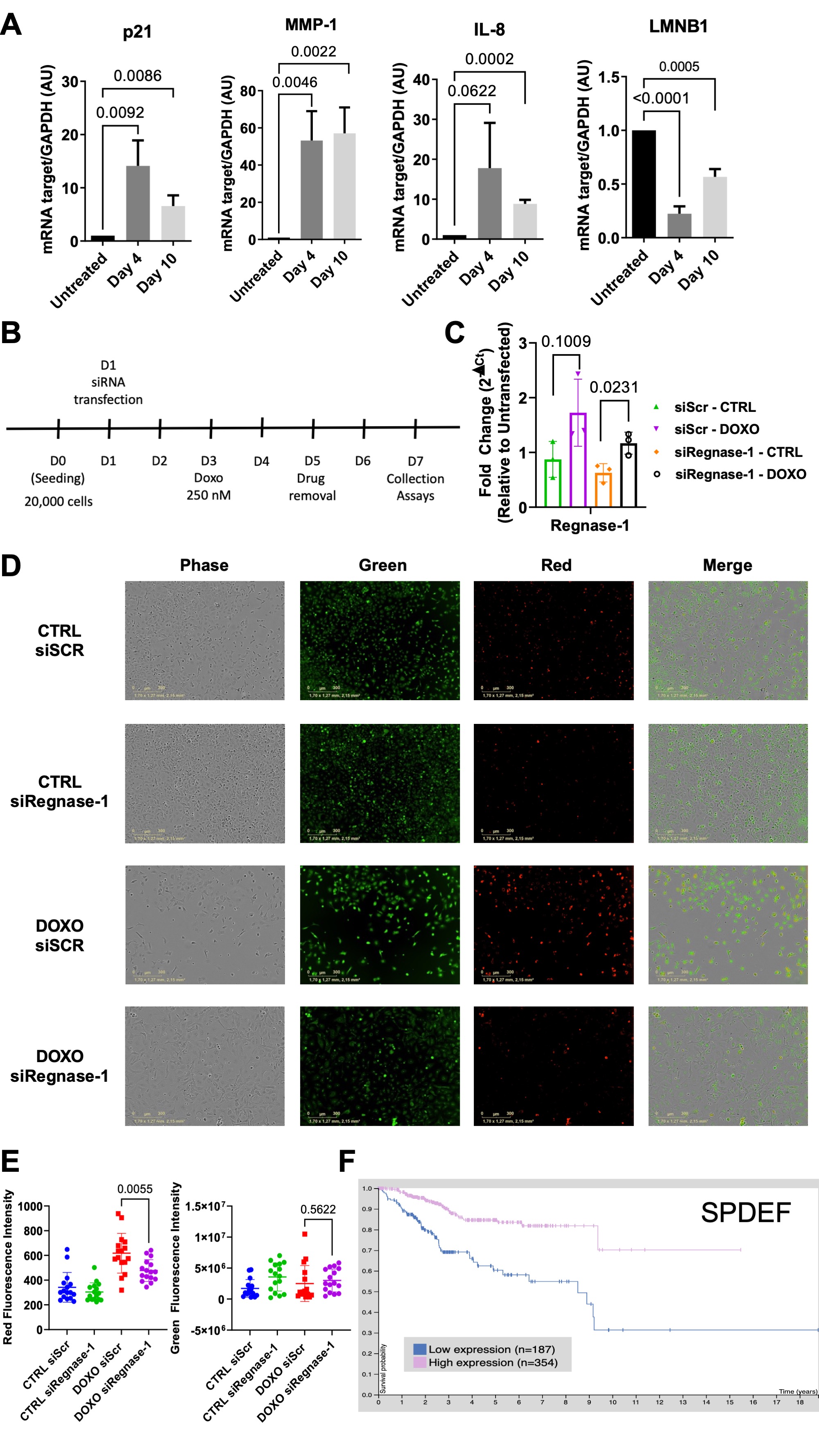

Supplement: Supplementary file 4 — Supplementary file4 (JPG 991 KB) [file 11357_2023_909_MOESM4_ESM.jpg]
